# Supplementary material for: A UK general practice population cohort study investigating the association between lipid lowering drugs and 30-day mortality following medically attended acute respiratory illness
Source: PeerJ. 2016 Apr 18;4:e1902. doi: 10.7717/peerj.1902 (PMC4841228; doi:10.7717/peerj.1902)
Supplement: Appendix S1 [file peerj-04-1902-s001.docx]

| **Variable name in Stata** | **Description** | **Coding and distribution of variables (all percentages rounded to the nearest decimal place)** |
| --- | --- | --- |
| patid | Unique patient identifier | String |
| **Patient socio-demographic and lifestyle** | | |
| Maari | Medically attended acute respiratory infection | 1= yes (201,179; 100%)  .= missing (0; 0%) |
| Death30 | Death 30 days following maari episode  Binary variable | 1= yes (1,096; 0.5%)  2= no (200,083; 99.5%) |
| Age | Age (at maari episode) in years; continuous variable | Median (IQR): 52 (41-66)  Missing: 0% |
| Gender | Sex  Binary variable | 0=male (82,070; 41%)  1=female (119,109; 59%)  .=missing (0%) |
| Bmi_threshold | Body mass index categorised as:underweight (<18.5), normal (18.5-24.9), overweight (25.0-29.9), obese (>30.0)  Categorical variable | 1= underweight (2,694; 1%)  2= normal (42,278; 21%)  3= overweight (44,337; 22%)  4= obese (33,278; 17%)  .= missing (78,592; 39%) |
| smokestatus | Smoking status  Ordered categorical variable | 1= never smoker (24,439; 12%)  2= ex-smoker (74,980; 37%)  3= current smoker (34,419; 17%)  .= missing (67,341; 33%) |
| Current_hba1c | Most recent measurement for glycated haemogloblin A1c by GP ( prior to MAARI episode)  Binary variable | 1= yes (5; 0%)  0= no (201,174; 100%)  .= missing (0; 0%) |
|  |  |  |
| **Disease Covariate Variables** | | |
| Hypten | Hypertension as defined and recorded by GP in CPRD  Binary variable | 1= yes (42,463; 21%)  0= no (158,716; 79%)  . = missing (0; 0%) |
| mi | Myocardial infarction as defined and recorded by GP in CPRD  Binary variable | 1= yes (7,088; 3.5%)  0= no (194,091; 96.5%)  . = missing (0; 0%) |
| hrtfl | Heart failure as defined and recorded by GP in CPRD  Binary variable | 1= yes (2,368; 1%)  0= no (198,811; 99%)  . = missing (0; 0%) |
| vasc | Peripheral vascular disease as defined and recorded by GP in CPRD  Binary variable | 1= yes (2,067; 1%)  0= no (199,112; 99%)  . = missing (0; 0%) |
| cld | Chronic lung disease as defined and recorded by GP in CPRD  Binary variable | 1= yes (37,728; 19%)  0= no (163,451; 81%)  . = missing (0; 0%) |
| diab | Diabetes as defined and recorded by GP in CPRD  Binary variable | 1= yes (19,063; 9%)  0= no (182, 116; 91%)  . = missing (0; 0%) |
| ccicat | Charlson’s comorbidity index scores;; grouped into 4 categories  Ordered categorical | 0= CCI score of zero (145,924; 73%)  1= CCI score 1-2 (39,964; 20%)  2= CCI score 3-5 (11,497; 6%)  3= CCI score 5 or greater (3,794; 2%)  . = missing (0; 0%) |
| **Drug Covariate Variables** | | |
| Current_statin | Current prescription for statins by GP (in30-days prior to MAARI episode)  Binary variable | 1= yes (27,095; 13.5%)  0= no (174, 084; 86.5%)  .= missing (0; 0%) |
| Current_fibrates | Current prescription for fibrates by GP (in30-days prior to MAARI episode)  Binary variable | 1= yes (611; 0.3%)  0= no (200,568; 99.7%)  .= missing (0; 0%) |
| Current_glitazones | Current prescription for glitazones by GP (in30-days prior to MAARI episode)  Binary variable | 1= yes (923; 0.5%)  0= no (200,256; 99.5%)  .= missing (0; 0%) |
| Current_metformin | Current prescription for metformin by GP (in30-days prior to MAARI episode)  Binary variable | 1= yes (6,617; 3%)  0= no (194,562; 97%)  .= missing (0; 0%) |
| Current_arb | Current prescription for angiotensin receptor blockers by GP (in30-days prior to MAARI episode)  Binary variable | 1= yes (8,145; 4%)  0= no (193,034; 96%)  .= missing (0; 0%) |
| Current_ace | Current prescription for angitensin converter enzyme by GP (in30-days prior to MAARI episode)  Binary variable | 1= yes (4,033; 2%)  0= no (197, 146; 98%)  .= missing (0; 0%) |
|  |  |  |
| Current_bb | Current prescription for beta-blockers by GP (in30-days prior to MAARI episode)  Binary variable | 1= yes (7,762; 4%)  0= no (193,417; 96%)  .= missing (0; 0%) |
