# Supplementary material for: A UK general practice population cohort study investigating the association between lipid lowering drugs and 30-day mortality following medically attended acute respiratory illness
Source: PeerJ. 2016 Apr 18;4:e1902. doi: 10.7717/peerj.1902 (PMC4841228; doi:10.7717/peerj.1902)
Supplement: Appendix S2 [file peerj-04-1902-s002.docx]

| Statin exposure models | | |  | |  |  | |  |
| --- | --- | --- | --- | --- | --- | --- | --- | --- |
| **Model** | Variables included in logistic regression and cox proportional hazards regression analysis | | | | | | | |
|  | **Exposure** | ***A priori*** | | **Disease covariates** | | | **Drug covariates** | **Socio-demographic and lifestyle** |
| **Model A** | statin | Age  Gender  Fibrates Metformin Glitazones | | Myocardial infarction  Hypertension  Peripheral vascular disease  Heart failure  Chronic lung disease  Diabetes | | | ACE inhibitors  Arb | Smoking status  Charlson’s comorbidity index  Body mass index  HbA1c |
| **Model B** | statin | Age  Gender  Fibrates Metformin Glitazones | | Myocardial infarction  Hypertension  Peripheral vascular disease  Heart failure  Chronic lung disease  Diabetes | | |  | Smoking status  Charlson’s comorbidity index  Body mass index  HbA1c |
| **Model C** | statin | Age  Gender  Fibrates Metformin Glitazones | | Myocardial infarction  Hypertension  Heart failure  Diabetes | | |  | Charlson’s comorbidity index  Body mass index |
