# Supplementary material for: A UK general practice population cohort study investigating the association between lipid lowering drugs and 30-day mortality following medically attended acute respiratory illness
Source: PeerJ. 2016 Apr 18;4:e1902. doi: 10.7717/peerj.1902 (PMC4841228; doi:10.7717/peerj.1902)
Supplement: Appendix S3 [file peerj-04-1902-s003.docx]

|  | | | | |
| --- | --- | --- | --- | --- |
| **Outcome measure: influenza-related mortality (30-day mortality)** | **Ratio of unexposed to exposed** | **Hazard ratio (unexposed v. Exposed)** | **Exposed patients** | **Unexposed patients** |
| **Exposure measure: statins** | 3 | 0.90 | 2,802 | 8,406 |
| **Exposure measure: fibrates** | 3 | 0.95 | 12,064 | 36,192 |
| Standard assumptions:  Powered at 80% with a type 1 error probability of 0.05  Median survival time in unexposed cases assumed to be 6 years (based on previous work in this topic area) with the ratio of unexposed to exposed subjects 3:1; accrual time of 5 years and follow-up of 1 year  Hazard ratios based on previous point estimates obtained from scientific literature and consultation with study collaborators taking into account clinical significance [[26](#_ENREF_26), [27](#_ENREF_27)] | | | | |
