# Supplementary material for: A UK general practice population cohort study investigating the association between lipid lowering drugs and 30-day mortality following medically attended acute respiratory illness
Source: PeerJ. 2016 Apr 18;4:e1902. doi: 10.7717/peerj.1902 (PMC4841228; doi:10.7717/peerj.1902)
Supplement: Appendix S4 [file peerj-04-1902-s004.docx]

| **Comparison of patient characteristics among survivors and non-survivors.** | | | | | |
| --- | --- | --- | --- | --- | --- |
| **Patient Characteristic** | **Survivors (n=200,083)** | **Non-Survivors (n=1,096 )** | **Unadjusted OR^1^ (95% Confidence Interval)** | | **P value^2^** |
| **Median Age (IQR)^3^** | 52(41-66) | 84 (75-89) | 1.12 (1.11 to 1.12) | **<0.001** | |
| **Sex** |  |  |  |  | |
| Males | 81,560 (40.8%) | 510 (46.5%) | 1 |  | |
| Females | 118,523 (59.2%) | 586 (53.5%) | 0.79 (0.74 to 0.90) | **<0.001** | |
| **Hypertension** | 42,013 (21.0%) | 450 (41.1%) | 2.62 (2.32 to 2.96) | **<0.001** | |
| **Myocardial Infarction** | 6,956 (3.5%) | 132 (3.5%) | 3.80 (3.16 to 4.57) | **<0.001** | |
| **Heart Failure** | 2,287 (1.1%) | 81 (7.4%) | 6.90 (5.49 to 8.69) | **<0.001** | |
| **Peripheral Vascular Disease** | 2,017 (1.0%) | 50 (1.0%) | 4.69 (3.52 to 6.26) | **<0.001** | |
| **Chronic Lung Disease** | 37,482 (18.7 %) | 246 (22.5 %) | 1.26 (1.19 to 1.45) | **0.002** | |
| **Diabetes** | 18,888 (9.4%) | 175 (16.0%) | 1.82 (1.55 to 2.14) | **<0.001** | |
| **Charlson’s Comorbidity Score** |  |  |  |  | |
| 0 | 145,642(72.8%) | 282 (25.7%) | 1 |  | |
| 1-2 | 39,454 (19.7%) | 510 (46.5%) | 6.68 (5.77 to 7.72) |  | |
| 3-5 | 11,277 (5.6%) | 220 (20.1%) | 10.18 (8.43 to 12.03) |  | |
| > 5 | 3,710 (1.9%) | 84 (7.7%) | 11.69 (9.15 to 14.95) | **<0.001** | |
| **Statins** | 26,882 (13.4%) | 213 (19.4%) | 1.55 (1.34 to1.81) | **<0.001** | |
| **Fibrates** | 607 (0.3%) | 4 (0.4%) | 1.20 (0.45 to 3.22) | 0.712 | |
| **Glitazones** | 920 (0.5%) | 3 (0.3%) | 0.59 (0.19 to 1.85) | 0.369 | |
| **Metformin** | 6,576 (3.3%) | 41 (3.7%) | 1.14 (0.34 to 1.57) | 0.401 | |
| **HbA1c** | 3 (0.0%) | 2 (0.2%) | 121.93 (20.36 to 730.40) | **<0.001** | |
| **ACE** | 4,004 (2.0%) | 29 (2.7%) | 1.33 (0.92 to 1.93) | 0.13 | |
| **Beta Blockers** | 7,692 (3.8%) | 70 (6.4%) | 1.71 (1.34 to 2.18) | **<0.001** | |
| **ARB** | 8,102 (4.1%) | 43 (3.9%) | 0.97 (0.71 to 1.31) | 0.833 | |
| **Smoking status** |  |  |  |  | |
| never smoker | 24,343 (18.3%) | 96 (13.3 %) | 1 |  | |
| ex-smoker | 74,636 (56.1%) | 344 (47.6%) | 1.17 (0.93 to 1.47) |  | |
| current smoker | 34,137 (25.6%) | 282 (39.1%) | 2.09 (1.66 to 2.64) | <0.001^*^ | |
| **BMI^4^** |  |  |  |  | |
| underweight | 2,587 (2.1%) | 107 (17.2%) | 1 |  | |
| normal weight | 42,004 (34.5%) | 247 (44.1%) | 1.15 (0.98 to 1.37) |  | |
| overweight | 44,189 (36.2%) | 148 (23.7%) | 1.54 (1.14 to 2.08) |  | |
| obese | 33,185 (27.2%) | 93 (15.0%) | 2.71 (1.56 to 4.72) | <0.001^*^ | |
| ^1^Odds ratio; ^2^Wald’s *p values*; ^3^Interquartile range; ^4^Body mass index; ^*^Wald’s *p value* for trend  Significant p values shown in bold | | | | | |
